# Supplementary material for: Defining a “Good Death” in Pediatric Oncology: A Mixed Methods Study of Healthcare Providers
Source: Children (Basel). 2020 Jul 31;7(8):86. doi: 10.3390/children7080086 (PMC7465817; doi:10.3390/children7080086)
Supplement: Supplementary file 1 [file children-07-00086-s001.pdf]

children

Box S1: Qualitative Interview Guide.

[*Prior to recording*]

Thank you for agreeing to participate in this project. Before we begin, I'd like to review a few important points:

1) Please say exactly what you think. Don't worry about what I think. This is about learning from your perspectives and experiences.
2) With that in mind, talk about your experience and feelings, not about what you have heard others say about this issue. Please express your opinions freely.
3) This conversation will be taped, so please do speak up and speak clearly.
4) Any reference to individual people or patients by name will be deleted from transcripts so that no identifiers are left in our research records. That said, identifying information will still remain on the audio file, which will be kept confidential until destroyed. Audio-recordings will be destroyed once analysis of all interview transcripts is completed.

Do you have any questions before we begin?

[*Begin Recording*]

Today, I want to talk about what constitutes a "good death." Much of what we think about this subject has come from studies of older adults; we don't know a whole lot about what is a "good death" for adolescents and young adults. We wonder what are the unique and common things that are important for people of this age and their families? We are interested in this because we want to try to improve people's experiences at the end of life. In order to do that, we need to understand what people feel are the important things that would make a death "good" or "bad." You may have been present for someone's death. Or, you may have thought about what is important at the end of life based on your own experiences. We are interested in finding out what kind of things you think make a death good or bad.

Do you have any thoughts before we begin?

[*Specific Questions]*

- First, let me start by asking, have you ever seen (or heard of) someone dying in a way you thought was particularly good or bad?
- Do you mind telling me about it?
- Do you think it was a good death?
- Do you think it was a bad death?
- Why? What made it good/bad?
- What kinds of things do you think would make a death a good death?

[*Probe Questions*]

- Would it be important not to have pain at the time of death?
- Who do you think should be present when adolescents or young adults die? If it were you, would you want to be alone or have others with you?
- How important is it to have friends and family nearby?
- What kind of role would you like your friends or family to have in your death?
- How important is it to have clergy/religious figures nearby?
- How important would it be to say goodbye to people?

- Where do you think it would be good to die? Would you prefer to die at home, at the hospital, or somewhere else?
- How do you feel about being sedated?
- How important would it be to know in advance what is likely to happen when you die?
- How can health care providers affect the quality of death?
- What do you think are the roles of nurses, doctors, social workers, or other staff in affecting the quality of death?
- Imagine if you were to measure good compared to bad deaths. Can you give two things you would measure?
- How much control do you want over your death?
- Do you think about how much money will be spent related to your death?
- What do you consider to be proper financial preparation related to your death? This might mean making wills and planning for a funeral.
- What are your feelings about the uncertainty that surrounds death?
- What are your feelings about the communication between the dying person, the person's family, and the person's caregivers around the time of death?
- What kinds of emotions do you think are normal around the time of dying?

Thank you for discussing these questions with me today.

[*Closing Questions*]

- How has this been for you?
- What support person, group or community will you reach out to if you feel anxious or sad after our conversation?
- Do you have any questions for me?
- Are there any additional resources that I can provide for you at this time?
